# Supplementary material for: Identification of photoperiod-induced specific miRNAs in the adrenal glands of Sunite sheep (Ovis aries)
Source: Front Vet Sci. 2022 Jul 22;9:888207. doi: 10.3389/fvets.2022.888207 (PMC9354845; doi:10.3389/fvets.2022.888207)
Supplement: Supplementary file 1 [file Data_Sheet_1.ZIP › Supplementary file/miRDeep2 file.zip!/miRDeep2 file/Novel_Structure/SP42A1/Novel_682.pdf]

Provisional ID	:	NC_019460.2_5585
Score total	:	6.4
Score for star read(s)	:	3.9
Score for read counts	:	-1.3
Score for mfe	:	2.3
Score for randfold	:	1.6
Score for cons. seed	:	
Total read count	:	9
Mature read count	:	8
Loop read count	:	0
Star read count	:	1

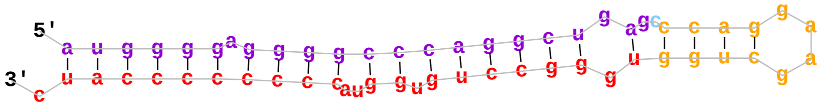

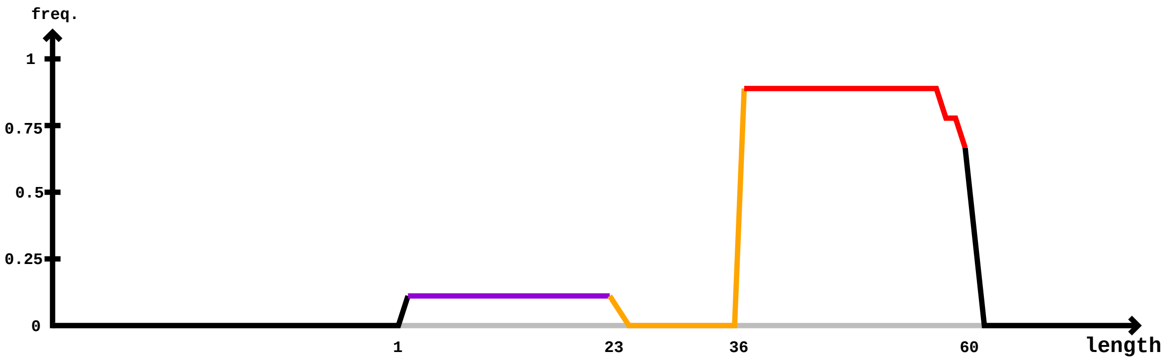


```
                                  Star                                Mature
5'- agaggaaguugaaugaggacuggaguggagguuggauggggaggggcccaggcugagcccaggaagcugguggggccugugguaccccccccauccaaggagaccagagucc   -3'   obs
    agaggaaguugaaugaggacuggaguggagguuggauggggaggggcccaggcugagcccaggaagcugguggggccugugguaccccccccauccaaggagaccagagucc         exp
    ................(((((...((.....((((((((((.((((((((((((.(..((((....))))).)))))).))..))))))))))))))....))...)))))   reads   mm   sample
    ...................................auggggaggggcccaggcugag......................................................   1       0    seq
    ......................................................................ugggccugugguacccccccc....................   1       0    seq
    ......................................................................ugggccugugguaccccccccau..................   1       0    seq
    ......................................................................ugggccugugguaccccccccauc.................   4       0    seq
    ......................................................................ugggccugugguaccccccccaucc................   2       0    seq
```
